# GPER介导的大气细颗粒物致肺癌作用研究进展

**DOI:** 10.3779/j.issn.1009-3419.2026.101.13

**Published:** 2026-06-20

**Authors:** Zhenhua LI, Dingbiao LI

**Affiliations:** 650051 昆明，昆明医科大学附属延安医院胸外科; Department of Thoracic Surgery, Yan'an Hospital Affiliated to Kunming Medical University, Kunming 650051, China

**Keywords:** 肺肿瘤, G蛋白偶联雌激素受体, 大气细颗粒物, 呼吸系统, Lung neoplasms, G protein-coupled estrogen receptor, Fine particulate matter, Respiratory system

## Abstract

肺癌是全球癌症相关死亡的首要原因，大气细颗粒物（fine particulate matter, PM_2.5_）已被列为I类人类致癌物，大量流行病学证明其与肺癌发生发展存在紧密联系。G蛋白偶联雌激素受体（G protein-coupled estrogen receptor, GPER）近年来被发现可被多种环境污染物激活并诱发肿瘤，且在肺癌组织中显著高表达，揭示其在肺癌进展中扮演重要角色。研究发现，PM_2.5_中的环境雌激素类组分、金属离子等可激活GPER，进而调控丝裂原活化蛋白激酶/细胞外信号调节激酶（mitogen-activated protein kinase/extracellular signal-regulated kinase, MAPK/ERK）、磷脂酰肌醇3-激酶/蛋白激酶B（phosphoinositide 3-kinase/protein kinase B, PI3K/AKT）等下游信号通路，诱导炎症反应、氧化应激反应和铁死亡，最终影响肿瘤细胞增殖、凋亡、迁移、上皮-间质转化，重塑肿瘤微环境，共同驱动肺癌的发生和恶性进展。总之，GPER作为连接PM_2.5_环境暴露与肺癌恶性进展的关键分子枢纽，是极具潜力的干预靶点，靶向GPER的抑制剂有望为肺癌防治提供新策略。

## Abstract (en)

Lung cancer remains the leading cause of cancer-related death worldwide. Fine particulate matter (PM_2.5_), classified as a group I human carcinogen, has been extensively linked to lung cancer development through epidemiological studies. The G protein-coupled estrogen receptor (GPER) can be activated and induce tumorigenesis by environmental pollutants, and it is significantly overexpressed in lung cancer tissues, highlighting its critical role in disease progression. Research indicates that environmental estrogen-like components and metal ions in PM_2.5_ can activate GPER, thereby regulating downstream signaling pathways such as mitogen-activated protein kinase/extracellular signal-regulated kinase (MAPK/ERK) and phosphoinositide 3-kinase/protein kinase B (PI3K/AKT). This activation triggers inflammatory responses, oxidative stress, and ferroptosis, ultimately promoting tumor cell proliferation, apoptosis, migration, epithelial-mesenchymal transition, and remodeling of the tumor microenvironment. Collectively, these mechanisms drive the initiation and progression of lung cancer. In summary, GPER serves as a pivotal molecular hub connecting PM_2.5_ exposure to lung cancer pathogenesis, presenting a promising therapeutic target. Inhibitors targeting GPER may offer novel strategies for the prevention and treatment of lung cancer.

肺癌是全球范围内发病率和死亡率最高的恶性肿瘤之一。统计数据^[1,2]^显示，肺癌发病数和死亡数分别占全球癌症总数的37.0%和39.8%，其中非小细胞肺癌（non-small cell lung cancer, NSCLC）占大多数。我国肺癌的发病率和死亡率均位居恶性肿瘤首位，是严重威胁公众健康的重要因素。研究^[3,4]^显示，2016年空气污染已成为仅次于吸烟致肺部非传染性疾病的第二风险因素，且在东南亚地区或成为肺癌最大病因，而大气细颗粒物（fine particulate matter, PM_2.5_）作为空气污染的主要成分，已被国际癌症研究机构列为I类人类致癌物，引起广泛关注。流行病学研究^[5]^表明，PM_2.5_暴露与肺癌风险、肺癌死亡率显著相关，其风险比（relative risk, RR）分别为1.16、1.23。随着工业化进程加速，人们越来越意识到PM_2.5_污染已成为全球重大的公共卫生挑战。G蛋白偶联雌激素受体（G protein-coupled estrogen receptor, GPER），又称G蛋白偶联受体30（G protein-coupled receptor 30, GPR30），作为一种新型膜受体，在肺癌组织中显著高表达，且与肿瘤分期晚、分化程度差密切相关^[6]^。近年来发现GPER能够介导多种环境污染物的雌激素样效应，导致人体内分泌与代谢紊乱，调节DNA损伤，诱发包括癌症在内的多种疾病，因此其也被称作“内分泌扰乱物受体”^[7,8]^。PM_2.5_中的雌激素样组分可能激活GPER，通过多途径驱动内环境改变促进肺癌发展。本文旨在系统综述GPER在PM_2.5_致肺癌中的作用机制，重点探讨其与PM_2.5_组分的交互网络、下游信号通路及肿瘤微环境重塑功能，为靶向GPER的肺癌防治策略提供新视角。

## 1 GPER的生物学特性及其在肺癌中的病理生理学作用

GPER是一种7次跨膜GPR，其基因位于染色体7p22.3，编码375个氨基酸。与经典雌激素受体（estrogen receptor alpha/beta, ERα/ERβ）不同，GPER主要定位于细胞膜和内质网，可通过非基因组信号通路快速响应雌激素刺激。研究^[9]^已证实，体内的多种类固醇样雌激素（如雌二醇、雌三醇等）和体外的大量雌激素样化合物（如双酚A、染料木黄酮等）均可激活GPER，引发人体代谢紊乱，导致心血管疾病和多种癌症形成等。GPER被激活后，可通过偶联刺激性G蛋白（stimulatory G protein, Gs）或抑制性G蛋白（inhibitory/other G protein, Gi/o），启动下游信号传导。具体而言，其激活可触发腺苷酸环化酶的活性变化，调节环磷酸腺苷（cyclic adenosine monophosphate, cAMP）的生成，进而影响细胞内钙离子动员^[10,11]^。同时，GPER还能通过Gβγ亚基反式激活表皮生长因子受体（epidermal growth factor receptor, EGFR），以调控丝裂原活化蛋白激酶/细胞外信号调节激酶（mitogen-activated protein kinase/extracellular signal-regulated kinase, MAPK/ERK）通路、磷脂酰肌醇3-激酶/蛋白激酶B（phosphoinositide 3-kinase/protein kinase B, PI3K/AKT）通路等重要信号转导途径。此外，GPER与ERα之间存在交叉对话，可协同调节靶基因的转录活性，共同影响细胞增殖、分化和存活等生物学过程^[12,13]^。

近年来多项研究^[6,14-17]^均证实GPER在肺癌组织中高表达，可表达于胞核、胞浆、胞膜；且不同的亚细胞定位与肿瘤分期、患者预后及ERβ表达密切相关。体外实验^[16]^表明，G1（GPER激动剂）处理可促进A549、H1793肺癌细胞增殖、迁移和侵袭，而使用G15（GPER拮抗剂）能逆转上述效应。在动物模型中，氨基甲酸酯诱导的肺腺癌小鼠经G1处理后，肿瘤质量和肿瘤结节数增加；而G15干预可抑制肿瘤生长。临床数据库分析进一步证实，GPER高表达与患者总生存期缩短相关。这些结果直接证明了GPER在肺癌发生中的促进作用。

## 2 PM_2.5_组分及促进肺癌发生的机制

PM_2.5_是大气污染的关键组分，其化学成分复杂多样，主要来源于化石燃料燃烧、工业排放、机动车尾气及生物质燃烧等一次排放物，以及大气中气态前驱物经光化学反应生成的二次气溶胶。从化学组成看，PM_2.5_包含有机碳、元素碳、无机盐、重金属及生物组分等，包括多环芳烃（polycyclic aromatic hydrocarbons, PAHs）及其衍生物、黑碳、硫酸盐、硝酸盐、铵盐、镍、铬、铅^[18]^。这些组分具有粒径小、比表面积大、易携带毒性物质等特质，能轻易穿透上呼吸道防御系统进入肺泡，并穿过肺泡-毛细血管屏障进入血液循环，对全身多系统造成不可估量的危害^[19]^。流行病学研究^[20,21]^显示，数小时至数天内的PM_2.5_短期暴露即可显著增加居民全因死亡风险，尤其是心血管和呼吸系统疾病死亡风险。例如，中国272个城市的数据表明，PM_2.5_浓度每升高10 μg/m³，全因死亡、心血管疾病死亡和呼吸系统疾病死亡风险分别增加0.22%、0.27%和0.29%，而在美国、加拿大、西班牙等地区，PM_2.5_每升高10 μg/m³，全因死亡风险分别增加1.58%、1.70%、1.96%^[22]^。特别需要注意的是，长期PM_2.5_暴露与肺癌发病率、死亡率存在显著关联。有研究^[23]^认为，PM_2.5_可通过诱导氧化应激、炎症反应及DNA损伤等机制破坏呼吸系统屏障功能，导致肺功能下降；同时，其组分中的PAHs和重金属可直接或间接激活致癌通路，从而促进肺癌发生、发展。此外，PM_2.5_还被认为与心血管事件、神经系统疾病及代谢紊乱有关，其对健康的危害存在脆弱人群差异，儿童、老年人及已有心肺疾病患者更易受影响，且持续性高浓度暴露将大幅提高对心率、血压的影响，威胁肺功能^[24]^。因此，PM_2.5_暴露与多项健康损害之间的关联已得到确认。

PM_2.5_促进肺癌发生的分子机制涉及多通路协同作用。首先，PM_2.5_诱导的氧化应激是其致病的关键导火索。如铁、铜等金属组分及一些PAHs被PM_2.5_吸附进入细胞后，可通过Fenton反应催化产生大量活性氧（reactive oxygen species, ROS）。而过量积累的ROS将逐步打破细胞内氧化还原平衡，随后引发大范围的大分子损伤，包括细胞膜的脂质过氧化对膜结构与功能的破坏、DNA被攻击后造成碱基修饰、链断裂等遗传物质损害、蛋白质构象与活性的影响^[25,26]^。例如，一项来自圣保罗大学的研究^[27]^显示，PM_2.5_暴露不仅会显著提升肺组织中8-氧代鸟嘌呤（8-oxodG）等氧化性DNA加合物的水平，还可引起肺、肝DNA中5-羟甲基胞嘧啶（5-hydroxymethylcytosine, 5-hmC）显著降低，这类突变若得不到及时修复，极有可能逐步累积并诱发基因突变，成为细胞恶性转化的潜在起点。其次，持续性的炎症反应进一步放大并延续损伤：PM_2.5_可被肺泡巨噬细胞及呼吸道上皮细胞识别，激活细胞内活化B细胞核因子-κB（nuclear factor-κB, NF-κB）和MAPK通路等关键信号通路^[28]^。这些通路的激活促使细胞释放如肿瘤坏死因子-α（tumor necrosis factor-α, TNF-α）、白细胞介素-6（interleukin-6, IL-6）等大量促炎因子，形成局部炎症风暴^[29]^。这种慢性的低度炎症状态不仅直接损伤肺组织，还通过维持增殖信号、抑制细胞凋亡、促进组织重塑等方式，逐步建立有利于肿瘤发生与发展的促瘤微环境，推动疾病向更深阶段发展。

## 3 GPER与PM_2.5_对肺癌的交互作用

### 3.1 GPER介导的PM_2.5_致肺癌信号网络

PM_2.5_作为复杂的环境污染物，可通过直接或间接方式调节GPER信号通路。首先，PM_2.5_携带的PAHs等外源性雌激素样物质，具有与雌二醇等相似的分子结构，可作为配体直接与胞内GPER结合。其次，PM_2.5_暴露也可能导致肺部局部微环境中内源性雌激素水平升高，与GPER结合后，引起其构象变化，激活与之偶联的异源三聚体G蛋白亚基Gαs、Gαi蛋白，进而启动快速的非基因组信号传导，发生GPER介导的EGFR反式激活的核心下游事件^[30,31]^。具体而言，GPER激活后通过异源三聚体G蛋白亚基复合物Gβγ促进基质金属蛋白酶（matrix metalloproteinase, MMP）-2、MMP-9等活化，裂解前体肝素结合性表皮生长因子样生长因子（pro-heparin-binding epidermal growth factor-like growth factor, pro-HB-EGF）等跨膜前体蛋白，释放出成熟的HB-EGF，进而与邻近细胞的EGFR结合，启动两条关键的致癌信号通路——MEK/ERK通路和PI3K/AKT通路^[32,33]^。这些通路被激活后，最终汇聚于细胞核，调控与细胞增殖、存活、迁移及上皮-间质转化（epithelial-mesenchymal transition, EMT）相关的基因，直接驱动肺癌细胞的恶性进展。大量流行病学与毒理学研究已证实，PM_2.5_是肺癌发生与发展的重要环境危险因素，而GPER在肺癌组织中存在异常高表达，理论上，PM_2.5_的致癌效应可能部分由GPER介导的信号通路所驱动，二者在肺癌进程中存在潜在的因果关联。

### 3.2 PM_2.5_通过甲基化修饰影响GPER表达致肺癌发生

PM_2.5_不仅通过激活GPER信号通路诱发肺癌，还可能通过DNA甲基化修饰影响GPER表达致肺癌发生。多项研究发现肺癌中*GPER*基因甲基化水平及DNA甲基转移酶（DNA methyltransferases, DNMTs）较正常肺组织显著升高^[34,35]^，而*GPER*甲基化可影响其自身转录水平并导致肿瘤形成^[36]^。具体而言，PM_2.5_通过肺泡进入血液循环后引发系统性炎症和氧化应激，进而通过多种途径干扰表观遗传修饰机制：首先，PM_2.5_诱导产生的大量ROS可改变DNMTs和DNA去甲基化酶TET（ten-eleven translocation）的催化活性，导致全基因组低甲基化或特定位点的高甲基化，例如，已在人支气管上皮细胞和肺腺癌细胞中发现PM_2.5_暴露可影响DNMTs和TET酶活性^[37,38]^；其次，PM_2.5_暴露可干扰一碳代谢循环，改变S-腺苷甲硫氨酸与S-腺苷同型半胱氨酸的比例，从而影响甲基供体的可用性；另外，PM_2.5_暴露引发的炎症信号通路（如NF-κB）也可间接调控表观遗传修饰酶的招募与功能^[39]^。综上，目前虽然缺乏PM_2.5_暴露导致*GPER*基因甲基化修饰致肺癌的直接证据，但间接证据支持两者之间存在潜在的生物学关联。

### 3.3 *GPER*基因多态性与PM_2.5_对肺癌风险的交互作用

现有的ER基因-环境交互研究多集中于ERα/ERβ，而针对膜受体GPER的专项分析尚未见报道。在乳腺癌的致病风险研究^[40]^中发现，ERα rs2046210的多态性与PM_2.5_暴露存在独立及联合效应。鉴于ERα/ERβ与GPER之间存在协同和拮抗效应，以及PM_2.5_可通过表观遗传修饰调控GPER的表达，而GPER的单核苷酸多态性可能进一步影响这一调控过程的效率^[41]^。因此，间接证据支持*GPER*基因的多态性与PM_2.5_暴露对肺癌风险可能存在联合效应。

### 3.4 两者对氧化应激、炎症反应的影响

PM_2.5_的致癌作用不仅通过直接损伤实现，更关键的是其引发的氧化应激与慢性炎症反应。例如，学者杨琳雪等^[42]^在观察太原市PM_2.5_对肺癌细胞A549的影响时发现，PM_2.5_暴露可使细胞内炎症因子IL-6、TNF-α表达升高，且炎症因子升高与PM_2.5_浓度成正比。再如de Oliveira等^[27]^的动物实验证实，PM_2.5_可增加小鼠DNA中8-oxodG含量，直接证明了PM_2.5_暴露引发氧化应激导致DNA损伤的能力。其内在机制可能与PM_2.5_的化学组分如铁、铜等金属离子能通过Fenton反应产生高活性的羟基自由基，导致脂质、蛋白、DNA等氧化损伤有关。值得注意的是，GPER在此过程中扮演了关键的信号整合与放大角色。一方面，GPER具有组成性活性，即在没有特异性激动剂的条件下，GPER信号依然可以激活NADPH氧化酶，催化超氧阴离子生成从而产生大量ROS^[43,44]^。另一方面，砷化物和镉等致癌金属可在肺癌细胞中通过GPER激活MAPK/ERK通路^[45,46]^。这种GPER-ERK1/2通路可在多种类型细胞中诱导或调节DNA损伤^[8,47]^。综上，GPER的激活作为PM_2.5_致癌的核心枢纽，通过精密调控氧化应激与炎症反应通路，间接而有力地加剧肺肿瘤的恶性进展。

### 3.5 二者对铁死亡的影响

铁死亡是一种以脂质过氧化物积累、抗氧化防御体系崩溃为特征的铁依赖性的程序性细胞死亡形式，在肺癌的发生、发展中扮演重要角色^[48]^。除经典的氧化应激途径外，PM_2.5_还可以通过直接干扰铁代谢促进铁死亡：PM_2.5_中富含的生物可利用性铁离子可穿过细胞膜进入胞内，通过调节铁调素表达，增加细胞内铁负载，而过载的铁离子可通过削弱铁输出蛋白（ferroportin, FPN）的抑制作用促进铁滞留^[49]^。这种铁超载的状态进一步影响转铁蛋白受体1（transferrin receptor 1, TfR1）、FPN表达平衡，共同构成促铁死亡的微环境。GPER则在此过程中发挥着“信号协同器”的作用，可与多种生长因子受体发生功能交互。研究^[50]^表明，GPER能与盘状结构域受体酪氨酸激酶1（discoidin domain receptor tyrosine kinase 1, DDR1）形成复合物，调控其磷酸化状态及下游信号传导，也可与胰岛素样生长因子1（insulin-like growth factor 1, IGF-1）/IGF-1R轴发生交叉对话，影响PI3K/AKT和MAPK通路活性。通过这些复杂的交互网络，GPER能间接调节溶质载体家族7成员11（solute carrier family 7 member 11, SLC7A11）、谷胱甘肽过氧化物酶4（glutathione peroxidase 4, GPX4）、长链脂酰辅酶A合成酶家族成员4（acyl-CoA synthetase long chain family member 4, ACSL4）等铁死亡核心效应分子的表达^[51]^。这些相互作用网络表明，GPER不仅作为激素感应器，更作为信号整合节点，在PM_2.5_扰乱铁稳态、促进铁死亡中发挥放大和协调作用，从而深刻影响肺癌的发生发展。

### 3.6 二者对肺癌性别二态性的影响

肺癌的性别二态性包括组织学类型、驱动基因类型等性别差异，其中最典型的为*EGFR*突变型肺腺癌在女性中高发^[52,53]^。目前已证明PM_2.5_暴露对女性肺癌的发病风险显著高于男性^[54]^；另外，PM_2.5_暴露还能促进*EGFR*突变型肺癌的形成：PM_2.5_进入肺泡后可被巨噬细胞识别并诱发炎症，后者释放的IL-1β能刺激携带*EGFR*突变的肺泡上皮II型细胞（alveolar epithelial type II cell, AT2 ）异常增生，最终形成*EGFR*突变型肺腺癌^[55]^。GPER在此过程中则可能通过塑造具有性别差异的免疫微环境来促进*EGFR*突变型或野生型肺腺癌的形成。首先，肺癌中GPER的亚细胞定位与*EGFR*突变状态和雌激素信号密切相关：当GPER表达于细胞核内时，其不仅与*EGFR*突变率正相关，还与ERα/ERβ表达正相关；而当GPER表达于细胞浆中时，其与EGFR野生型频率正相关，与ERα/ERβ表达负相关^[17,33]^。 其次，GPER参与调节肺癌免疫微环境，其在表达水平上与肿瘤组织中单核细胞、静息态肥大细胞浸润正相关，而与浆细胞、M1型巨噬细胞、γδ T细胞浸润负相关^[56]^。另外，GPER在外周B细胞、T细胞以及单核细胞、嗜酸性粒细胞和中性粒细胞中均有表达^[57]^。综上，可以合理推测在女性体内高雌激素水平以及PM_2.5_携带的PAHs等外源性雌激素样物质的共同作用下，GPER可能通过改变在亚细胞内的定位并发挥差异功能，从而塑造具有性别差异的免疫微环境，最终导致肺癌的性别二态性。

### 3.7 二者在肿瘤微环境重塑中的作用

PM_2.5_通过GPER影响肿瘤微环境以促进肺癌发生发展的机制是一个多环节、网络化的复杂过程，主要涉及包括细胞增殖、凋亡、血管生成、侵袭转移以及免疫微环境重塑等关键生物学事件。在细胞增殖与凋亡方面，PM_2.5_激活GPER后，通过EGFR/MAPK通路上调细胞周期蛋白D1和周期蛋白依赖性激酶4等细胞周期蛋白表达，从而推动G_1_期进入S期，加速细胞分裂、促进增殖。与此同时，GPER还通过调控circNOTCH1/m6A甲基化NOTCH1信号通路来调节肺癌细胞增殖，这一发现首次将GPER与RNA表观遗传修饰和环状RNA联系起来^[15]^。此外，在调控基因转录层面，GPER通过c-Fos和c-Jun转录因子激活激活蛋白-1（activating protein-1, AP-1），促进一系列细胞增殖相关基因转录。动物实验进一步证实其促增殖作用，PM_2.5_与GPER激动剂G1均可增加肺结节数，并伴随Ki-67阳性率上升，直接支持了GPER、PM_2.5_在异常驱动增生中的核心角色。血管生成是肿瘤进展的关键，在PM_2.5_诱导的缺氧微环境中，GPER通过缺氧诱导因子-1α/血管内皮生长因子（hypoxia inducible factor-1α/vascular endothelial growth factor, HIF-1α/VEGF）轴促血管新生，进而上调关键促血管生成因子VEGF表达，使得肿瘤组织周围产生大量新生血管，为肿瘤生成提供有利环境，这一点在Tirado-Garibay等^[58]^关于肺癌模型显示GPER抑制剂减少微血管密度的研究中得到充分验证。此外，在肿瘤的转移与侵袭环节，二者与癌细胞的机械力传导和EMT过程紧密关联：PM_2.5_通过GPER调控Slug和Snail转录因子，下调上皮细胞标志物E-钙黏蛋白、上调间质细胞标志物波形蛋白，激活RhoA通路，调节癌细胞的收缩、侵袭和迁移^[59]^。另一方面，PM_2.5_激活GPER可诱导长链非编码RNA或环状RNA，通过复杂的分子网络加速侵袭行为。免疫微环境重塑也是GPER发挥作用的核心区域，PM_2.5_不仅通过GPER促进肿瘤相关巨噬细胞向M2型极化，同时上调程序性细胞死亡配体-1（programmed cell death-ligand 1, PD-L1）表达，使得T细胞功能受到抑制，助益免疫逃逸^[56,60]^。GPER在肺癌干细胞（lung cancer stem cells, LCSCs）中高表达，可能通过BAD磷酸化增强细胞存活，赋予化疗耐药性。最终，上述这些机制相互交织、协同作用，共同构成了一个由GPER介导的、PM_2.5_驱动的促癌网络。总而言之，GPER作为连接PM_2.5_与肺癌发生发展的核心分子枢纽，覆盖了增殖、凋亡、血管生成、侵袭等多维度信号网络，共同驱动肺癌的发生和恶性进展（图1）。

**图 1:**
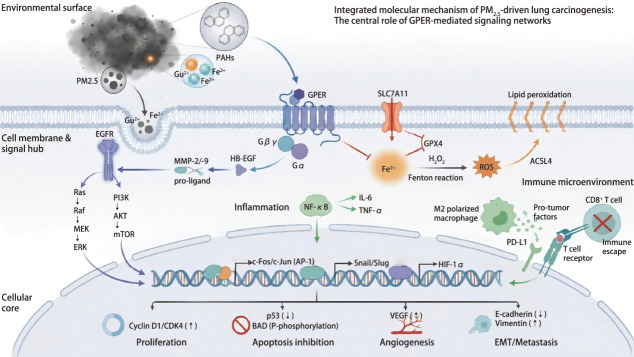
G蛋白偶联雌激素受体介导大气细颗粒物驱动肺癌发生的分子机制

## 4 小结与展望

GPER的发现及与PM_2.5_环境暴露的关联为理解肺癌的发生发展开启了一扇全新的窗口。本文梳理的现有证据表明，GPER不仅是经典雌激素信号通路的补充，更是一个独立且活跃的致癌信号枢纽。它通过整合内源性雌激素信号及外源性环境污染物如PM_2.5_等刺激信号，激活包括EGFR/MAPK、PI3K/AKT在内的多条下游通路，调控炎症反应，塑造免疫微环境，精密调控肿瘤细胞的增殖、凋亡、代谢重编程、血管新生及免疫逃逸，从而在肺癌的启动、演进和转移中扮演了核心驱动角色。然而，从基础研究走向临床转化，GPER靶向策略的前景虽令人期待，道路却充满挑战。

展望未来，该领域的突破有赖于在以下3个层面实现深度整合与创新。首先，在基础机制层面，当前研究大多分别描绘了PM_2.5_的毒性图谱和GPER的信号网络，二者间直接的、时空动态的因果链条仍显模糊。利用条件性基因敲除动物模型，在可控的PM_2.5_暴露场景下，必须明确GPER是必要中介因素还是协同因素，并解析其与铁死亡等新兴分子事件的交互网络。其次，在探究PM_2.5_暴露诱发的肺癌性别差异层面，除了关注GPER表达水平外，还应重点研究其亚细胞定位与肿瘤微环境中的雌激素水平和免疫微环境的关系，这将为GPER介导PM_2.5_驱动肺癌发生的机制以及肺癌性别二态性提供更全面的理解。同时，GPER的“双刃剑”效应提示，任何治疗策略都必须与肿瘤的分子分型及特定的肿瘤微环境特征相结合；未来的药物研发，不应仅仅聚焦于阻断GPER通路，还应关注激活该通路的效应，如美国生物技术公司在靶向GPER方面取得了进展，他们正在开展I/II期临床试验（NCT04130516）^[61]^，旨在评估GPER新型口服激动剂LNS8801在多种实体瘤中的疗效与安全性。此外，应在肺癌中探索靶向GPER与免疫治疗、化疗的协同方案。最后，在公共卫生层面，推动前沿发现向预防前移。通过大规模前瞻性队列研究，阐明*GPER*基因多态性与PM_2.5_暴露对肺癌风险的交互作用，有望识别出对环境致癌物尤其易感的高危人群，从而实现从“群体防护”到“个体化预警”的跨越。

综上所述，GPER代表了一个连接“环境暴露-细胞感知-肿瘤命运”的桥梁分子。尽管将其转化为有效的临床干预手段仍面临诸多科学和技术挑战，但其独特的生物学地位和明确的通路可及性，使其成为一个极具潜力的治疗靶点。通过深化机制理解、统一技术规范、创新药物设计并拓展预防应用，靶向GPER有望为肺癌的防治，特别是应对环境污染相关的肺癌负担，提供一条充满希望的新途径。这条路径的实现，最终需要基础科学家、临床医生和公共卫生专家的紧密协作，共同推动这一领域从实验室走向临床，惠及患者。

## References

[1] Li M, Hu M, Jiang L, et al. Trends in cancer incidence and potential associated factors in China. JAMA Network Open, 2024, 7(10): e2440381. doi: 10.1001/jamanetworkopen.2024.40381 39432306 PMC11581522

[2] Sun KX, Li L, Wang SM, et al. Cancer incidence and mortality across diverse geographical regions in China, 2024. Zhonghua Zhongliu Zazhi, 2026, 48(3): 400-412. 41876198 10.3760/cma.j.cn112152-20260205-00079

[3] Prüss-Ustün A, van Deventer E, Mudu P, et al. Environmental risks and non-communicable diseases. BMJ, 2019, 364: 1265. doi: 10.1136/bmj.l265 PMC6348403 30692085

[4] Xin WY, Guo XL, He XS, et al. Epidemiological trends and projections of lung cancer deaths levels and disease burden in Chinese residents, 1990-2019. Zhonghua Zhongliu Fangzhi Zazhi, 2024, 31(2): 65-73.

[5] Yu P, Guo S, Xu R, et al. Cohort studies of long-term exposure to outdoor particulate matter and risks of cancer: A systematic review and *meta*-analysis. Innovation (Camb), 2021, 2(3): 100143. doi: 10.1016/j.xinn.2021.100143 34557780 PMC8454739

[6] Jala VR, Radde BN, Haribabu B, et al. Enhanced expression of G-protein coupled estrogen receptor (GPER/GPR30) in lung cancer. BMC Cancer, 2012, 12: 624. doi: 10.1186/1471-2407-12-624 23273253 PMC3557142

[7] Qie Y, Qin W, Zhao K, et al. Environmental estrogens and their biological effects through GPER mediated signal pathways. Environ Pollut, 2021, 278: 116826. doi: 10.1016/j.envpol.2021.116826 33706245

[8] Lei B, Lu X, Yang Y, et al. Effects of two typical organophosphate flame retardants on DNA damage of GT1-7 cells and relevant molecular mechanism. Toxicology, 2025, 516: 154192. doi: 10.1016/j.tox.2025.154192 40389058

[9] Arterburn JB, Prossnitz ER. G protein-coupled estrogen receptor GPER: Molecular pharmacology and therapeutic applications. Annu Rev Pharmacol Toxicol, 2023, 63: 295-320. doi: 10.1146/annurev-pharmtox-031122-121944 36662583 PMC10153636

[10] Tran QK. Reciprocality between estrogen biology and calcium signaling in the cardiovascular system. Front Endocrinol (Lausanne), 2020, 11: 568203. doi: 10.3389/fendo.2020.568203 33133016 PMC7550652

[11] Li Q, Yang Y, Wang H, et al. Genistein accelerates glucose catabolism via activation the GPER-mediated cAMP/PKA-AMPK signaling pathway in broiler chickens. Life Sci, 2022, 303: 120676. doi: 10.1016/j.lfs.2022.120676 35640778

[12] Abbas MA, Al-Kabariti AY, Sutton C. Comprehensive understanding of the role of GPER in estrogen receptor-alpha negative breast cancer. J Steroid Biochem Mol Biol, 2024, 241: 106523. doi: 10.1016/j.jsbmb.2024.106523 38636681

[13] Pepermans RA, Sharma G, Prossnitz ER. G protein-coupled estrogen receptor in cancer and stromal cells: Functions and novel therapeutic perspectives. Cells, 2021, 10(3): 672. doi: 10.3390/cells10030672 33802978 PMC8002620

[14] Natale CA, Seykora JT, Ridky TW. Analysis of human GPER expression in normal tissues and select cancers using immunohistochemistry. bioRxiv, 2023, 10: 2023.03.09.531931. doi: 10.1101/2023.03.09.531931

[15] Shen Y, Li C, Zhou L, et al. G protein-coupled oestrogen receptor promotes cell growth of non-small cell lung cancer cells via YAP1/QKI/circNOTCH1/m6A methylated NOTCH1 signalling. J Cell Mol Med, 2021, 25(1): 284-296. doi: 10.1111/jcmm.15997 33237585 PMC7810948

[16] Liu C, Liao Y, Fan S, et al. G-protein-coupled estrogen receptor antagonist G15 decreases estrogen-induced development of non-small cell lung cancer. Oncol Res, 2019, 27(3): 283-292. doi: 10.3727/096504017X15035795904677 28877783 PMC7848463

[17] Li ZH, Liu C, Liu QH, et al. Cytoplasmic expression of G protein-coupled estrogen receptor 1 correlates with poor postoperative prognosis in non-small cell lung cancer. J Thorac Dis, 2022, 14(5): 1466-1477. doi: 10.21037/jtd-22-29 35693608 PMC9186222

[18] Zhong X, Sun LH, Chen H, et al. Short-term effects of PM_2.5_, O_3_ and CO on lung cancer mortality among residents in Pudong New Area. Zhonghua Zhongliu Fangzhi Zazhi, 2024, 31(21): 1285-1290.

[19] Li C, Wang JY, Zhao H. Trends of atmospheric composite pollution and human health effects in the Pearl River Delta region of Guangdong, Hong Kong and Mac. Huanjing Kexue Yanjiu, 2024, 37(6): 1378-1388.

[20] Sun FX, Xiong LL, Yang HF, et al. Assessing the excess mortality related to short-term exposure to PM_2.5_ in Nanjing from 2013 to 2019. Zhonghua Jibing Kongzhi Zazhi, 2021, 25(11): 1257-1263.

[21] Lu Y, Mao Y, Liu W, et al. Spatiotemporal trends of ischemic stroke burden attributable to PM_2.5_ from 1990 to 2021. Front Public Health, 2025, 13: 1608086. doi: 10.3389/fpubh.2025.1608086 40740382 PMC12307462

[22] Expert consensus writing group on acute health risks and prevention and control recommendations of atmospheric fine particulate matter in China, Environmental health branch of Chinese preventive medicine association, Health branch of the Chinese medical association. Acute health risks and prevention and control suggestions of atmospheric fine particulate matter in China. Zhonghua Yixue Zazhi, 2022, 102(18): 1341-1350.

[23] Yu H, Guo XY, Feng Q, et al. Health risk assessment based on specific sources of trace elements in PM_2.5_ from online observations. Huanjing Kexue Xuebao, 2025, 45(7): 104-112.

[24] Zhao ZM, Liu LL, Tian X, et al. Association between exposure to PM_2.5_ and chronic kidney disease in a population with metabolic syndrome. Weisheng Yanjiu, 2024, 53(3): 427-434. 38839584 10.19813/j.cnki.weishengyanjiu.2024.03.013

[25] Su X, Han CQ. Study on the mechanism of Hongjing Qingfei Formula on inflammation and oxidative stress induced by PM_2.5_ in mice. Shizhen Guoyi Guoyao, 2021, 32(2): 331-333.

[26] Xia R, Fang N, Yang Y, et al. PM_2.5_ promotes apoptosis of alveolar epithelial cells via targeting ROS/p 38 signaling pathway and thus leads to emphysema in mice. Minerva Med, 2023, 114(5): 652-657. doi: 10.23736/S0026-4806.20.06652-5 32491296

[27] de Oliveira AAF, de Oliveira TF, Dias MF, et al. Genotoxic and epigenotoxic effects in mice exposed to concentrated ambient fine particulate matter (PM_2.5_) from São Paulo city, Brazil. Part Fibre Toxicol, 2018, 15(1): 40. doi: 10.1186/s12989-018-0276-y 30340610 PMC6194750

[28] Hu L, Xu C, Tang X, et al. Fine particulate matter promotes airway inflammation and mucin production by activating endoplasmic reticulum stress and the IRE1α/NOD1/NF-κB pathway. Int J Mol Med, 2023, 52(4): 96. doi: 10.3892/ijmm.2023.5299 37654182 PMC10555484

[29] Wang BR, Shi JQ, Ge NN, et al. PM_2.5_ exposure aggravates oligomeric amyloid beta-induced neuronal injury and promotes NLRP 3 inflammasome activation in an *in vitro* model of Alzheimer’s disease. J Neuroinflammation, 2018, 15(1): 132. doi: 10.1186/s12974-018-1178-5 PMC5932821 29720213

[30] Abo-Zaid OA, Moawed FS, Hassan HA, et al. Bisphenol-A/radiation mediated inflammatory response activates EGFR/KRAS/ERK1/2 signaling pathway leads to lung carcinogenesis incidence. Int J Immunopathol Pharmacol, 2022, 36: 3946320221092918. doi: 10.1177/03946320221092918 PMC9009141 35410520

[31] Liu X, Yang Y, Tang X, et al. Shikonin mediates apoptosis through G protein-coupled estrogen receptor of ovarian cancer cells. Evid Based Complement Alternat Med, 2022, 2022: 6517732. doi: 10.1155/2022/6517732 36248433 PMC9556250

[32] Torres-Alamilla P, Castillo-Sanchez R, Cortes-Reynosa P, et al. Bisphenol A increases the size of primary mammary tumors and promotes metastasis in a murine model of breast cancer. Mol Cell Endocrinol, 2023, 575: 111998. doi: 10.1016/j.mce.2023.111998 37414130

[33] Li ZH, Pan Y, Liu Q, et al. Role of GPER1 in the mechanism of EGFR-TKIs resistance in lung adenocarcinoma. Front Oncol, 2022, 12: 869113. doi: 10.3389/fonc.2022.869113 35664735 PMC9158128

[34] Rong J, Xie X, Niu Y, et al. Correlation between the RNA expression and the DNA methylation of estrogen receptor genes in normal and malignant human tissues. Curr Issues Mol Biol, 2024, 46(4): 3610-3625. doi: 10.3390/cimb46040226 38666956 PMC11049367

[35] Fan YC, Wu W, Leng XF, et al. Utility of G protein-coupled oestrogen receptor 1 as a biomarker for pan-cancer diagnosis, prognosis and immune infiltration: A comprehensive bioinformatics analysis. Aging (Albany NY), 2023, 15(21): 12021-12067. doi: 10.18632/aging.205162 37921845 PMC10683611

[36] Pal U, Ghosh S, Limaye AM. DNA methylation in the upstream CpG island of the GPER locus and its relationship with GPER expression in colon cancer cell lines. Mol Biol Rep, 2020, 47(10): 7547-7555. doi: 10.1007/s11033-020-05817-5 32936384

[37] Li R, Zhao C, Zhang Y, et al. PM_2.5_-induced DNA oxidative stress in A 549 cells and regulating mechanisms by GST DNA methylation and Keap1/Nrf2 pathway. Toxicol Mech Methods, 2024, 34(5): 517-526. doi: 10.1080/15376516.2024.2307967 38293967

[38] Wang B, Li R, Cai Y, et al. Alteration of DNA methylation induced by PM_2.5_ in human bronchial epithelial cells. Toxicol Res (Camb), 2020, 9(4): 552-560. doi: 10.1093/toxres/tfaa061 32905279 PMC7467236

[39] Sun Q, Ren X, Sun Z, et al. The critical role of epigenetic mechanism in PM_2.5_-induced cardiovascular diseases. Genes Environ, 2021, 43(1): 47. doi: 10.1186/s41021-021-00219-w 34654488 PMC8518296

[40] Liaw YC, Wu CD, Liaw YC, et al. PM_2.5_ exposure and breast cancer risk: Independent and combined effects of *ESR1* rs2046210 and *FGFR2* rs 2981582 in a Taiwanese cohort. Environ Pollut, 2026, 399: 128142. doi: 10.1016/j.envpol.2026.128142 42002130

[41] Wu H, Eckhardt CM, Baccarelli AA. Molecular mechanisms of environmental exposures and human disease. Nat Rev Genet, 2023, 24(5): 332-344. doi: 10.1038/s41576-022-00569-3 36717624 PMC10562207

[42] Yang LX, Yang J, Wu XY, et al. Mechanism of A549 cell apoptosis induced by atmospheric fine particulate matter collected in Taiyuan city. Huanjing Yu Jiankang Zazhi, 2025, 42(4): 283-287.

[43] Meyer MR, Barton M. GPER blockers as Nox downregulators: A new drug class to target chronic non-communicable diseases. J Steroid Biochem Mol Biol, 2018, 176: 82-87. doi: 10.1016/j.jsbmb.2017.03.019 28343901

[44] Ishii T, Warabi E. Mechanism of rapid nuclear factor-E2-related factor 2 (Nrf2) activation via membrane-associated estrogen receptors: Roles of NADPH oxidase 1, neutral sphingomyelinase 2 and epidermal growth factor receptor (EGFR). Antioxidants (Basel), 2019, 8(3): 69. doi: 10.3390/antiox8030069 30889865 PMC6466580

[45] Huff MO, Todd SL, Smith AL, et al. Arsenite and cadmium activate MAPK/ERK via membrane estrogen receptors and G-protein coupled estrogen receptor signaling in human lung adenocarcinoma cells. Toxicol Sci, 2016, 152(1): 62-71. doi: 10.1093/toxsci/kfw064 27071941

[46] Boffetta P, Zaridze D, Świątkowska B, et al. Combined occupational exposure to carcinogenic metals/metalloids and risk of lung cancer. Front Oncol, 2026, 16: 1772676. doi: 10.3389/fonc.2026.1772676 42016703 PMC13093972

[47] Kozieł-Leszczyńska MJ, Habrowska-Górczyńska DE, Leszczyński KP, et al. AOH induces oxidative stress and DNA damage in ovarian cancer cells via modulation of GPER1 and HIF1α/PI3K/CLDNs signaling pathway. Sci Rep, 2026, 16(1): 3045. doi: 10.1038/s41598-026-36730-9 41571868 PMC12827960

[48] Ding JB, Kong DQ, Huang HM, et al. PM_2.5_ exposures exacerbate bleomycin-induced idiopathic pulmonary fibrosis in mice by regulating ferroptosis via Nrf2/SLC7A11/GPX4 axis. Zhongguo Yaolixue Tongbao, 2025, 41(2): 333-339.

[49] Yang L, Qiao Y, Huang Z, et al. Role of environmental pollutants-induced ferroptosis in pulmonary diseases. Front Med (Lausanne), 2025, 12: 1542275. doi: 10.3389/fmed.2025.1542275 40066170 PMC11891068

[50] Chen J, Zhao R, Wang Y, et al. G protein-coupled estrogen receptor activates PI3K/AKT/mTOR signaling to suppress ferroptosis via SREBP1/SCD1-mediated lipogenesis. Mol Med, 2024, 30(1): 28. doi: 10.1186/s10020-023-00763-x 38383297 PMC10880371

[51] Han ZW, Guo JS, Wang XC, et al. G protein-coupled estrogen receptor alleviates lung injury in mice with exertional heat stroke by inhibiting ferroptosis. Zhonghua Weizhongbing Jijiu Yixue, 2025, 37(3): 268-274. 10.3760/cma.j.cn121430-20241030-00896 40201998

[52] May L, Shows K, Nana-Sinkam P, et al. Sex differences in lung cancer. Cancers (Basel), 2023, 15(12): 3111. doi: 10.3390/cancers15123111 37370722 PMC10296433

[53] Yang Q, Zhang H, Wei T, et al. Single-cell RNA sequencing reveals the heterogeneity of tumor-associated macrophage in non-small cell lung cancer and differences between sexes. Front Immunol, 2021, 12: 756722. doi: 10.3389/fimmu.2021.756722 34804043 PMC8602907

[54] Myers R, Brauer M, Dummer T, et al. High-ambient air pollution exposure among never smokers versus ever smokers with lung cancer. J Thorac Oncol, 2021, 16(11): 1850-1858. doi: 10.1016/j.jtho.2021.06.015 34256112

[55] Hill W, Lim EL, Weeden CE, et al. Lung adenocarcinoma promotion by air pollutants. Nature, 2023, 616(7955): 159-167. doi: 10.1038/s41586-023-05874-3 37020004 PMC7614604

[56] Li Z, Xie C, Cui J, et al. GPER1 is involved in shaping tumor immune microenvironment and its expression is decreased in NSCLC tumorigenesis. Sci Rep, 2025, 15(1): 29488. doi: 10.1038/s41598-025-15405-x 40796933 PMC12343848

[57] Notas G, Kampa M, Castanas E, et al. G protein-coupled estrogen receptor in immune cells and its role in immune-related diseases. Front Endocrinol (Lausanne), 2020, 11: 579420. doi: 10.3389/fendo.2020.579420 33133022 PMC7564022

[58] Tirado-Garibay AC, Falcón-Ruiz EA, Ochoa-Zarzosa A, et al. GPER: An estrogen receptor key in metastasis and tumoral microenvironments. Int J Mol Sci, 2023, 24(19): 14993. doi: 10.3390/ijms241914993 37834441 PMC10573234

[59] Rice A, Cortes E, Lachowski D, et al. GPER activation inhibits cancer cell mechanotransduction and basement membrane invasion via RhoA. Cancers (Basel), 2020, 12(2): 289. doi: 10.3390/cancers12020289 31991740 PMC7073197

[60] Qing BM, Li XL, Ran Q, et al. PM_2.5_-induced M2 polarization and IL-1α secretion by tumor-associated macrophages promotes lung adenocarcinoma progression. Zhongguo Feiai Zazhi, 2025, 28(9): 667-679. 41309249 10.3779/j.issn.1009-3419.2025.102.33 PMC12666422

[61] Linnaeus Therapeutics Inc. Study assessing MTD, safety, tolerability, PK and anti-tumor effects of LNS 8801 alone and with Pembrolizumab. https://classic.clinicaltrials.gov/ct2/show/NCT04130516https://classic.clinicaltrials.gov/ct2/show/NCT04130516.Accessed December 18,2025.

